# Deciphering the Underlying Mechanisms Linking Psoriasis and IgA Nephropathy

**DOI:** 10.1155/jimr/1862174

**Published:** 2026-05-27

**Authors:** Zijie Tang, Jintong Wu, Meihan Dong, Chengxin Li, Rui Wang

**Affiliations:** ^1^ Medical School of Chinese People’s Liberation Army (PLA), Beijing, China; ^2^ Department of Dermatology, The First Medical Center of Chinese PLA General Hospital, Beijing, China, 301hospital.com.cn; ^3^ Department of Dermatology, Central Medical Branch of Chinese PLA General Hospital, Beijing, China; ^4^ State Key Laboratory of Kidney Diseases, The First Medical Center of Chinese PLA General Hospital, Beijing, China, 301hospital.com.cn

**Keywords:** chronic kidney disease, IgA nephropathy, interleukin, mechanism, psoriasis, signaling, tonsillitis, tumor necrosis factor

## Abstract

Psoriasis is a chronic, immune‐mediated inflammatory disease with systemic manifestations that include renal complications. Immunoglobulin A nephropathy (IgAN) represents the most common autoimmune glomerular disease worldwide. Growing epidemiological and genetic evidence supports a clinically relevant association between psoriasis and IgAN, particularly in patients with moderate‐to‐severe or arthropathic psoriasis. However, current knowledge remains limited by heterogeneous observational data and the absence of systematic renal screening in most psoriasis clinical trials. This review synthesizes evidence linking psoriasis and IgAN across multiple levels. Mendelian randomization studies point to a shared genetic basis, and transcriptomic analyses have uncovered overlapping immune signatures. Several inflammatory pathways may drive the production of galactose‐deficient IgA1 (Gd‐IgA1) and facilitate glomerular immune complex deposition. Aberrant IgA1 glycosylation driven by psoriatic inflammation, together with tonsillar immune activation, is proposed as a key connection between skin and kidney disease. Important knowledge gaps persist. Routine urinalysis is not performed in most psoriasis trials. Tonsillectomy data derive largely from East Asian populations, and no large‐scale international registries are currently available. We therefore propose a clinical screening framework and advocate for the establishment of dedicated registries, along with the integration of urinary tests into future trials. Addressing these gaps will enable mechanism‐based, personalized management of psoriasis‐associated IgAN.

## 1. Introduction

Psoriasis is a systemic inflammatory disease related to various autoimmune and inflammatory diseases, including renal complications [1, 2]. Epidemiological studies have demonstrated that chronic kidney disease (CKD) and end‐stage renal disease (ESRD) occur approximately two to three times more frequently in individuals afflicted with psoriasis [3–5]. The pathophysiological link between psoriatic skin inflammation and renal involvement remains incompletely understood, but recent experimental evidence suggests that systemic inflammation and immune dysregulation play pivotal roles. In a murine model of psoriasis induced by keratinocyte‐specific Tie2 overexpression (KC‐Tie2), chronic skin inflammation was associated with albuminuria, glomerulosclerosis, and elevated blood pressure [6]. Besides, systemic inflammation in psoriasis is also linked to oxidative stress and endothelial dysfunction, factors known to contribute to renal and cardiovascular complications [7]. These findings underscore the necessity of vigilant renal monitoring and comprehensive management of systemic inflammation in psoriatic patients to prevent or mitigate kidney involvement.

The association between psoriasis and CKD, particularly immunoglobulin A nephropathy (IgAN), has garnered attention in recent years due to the rising prevalence of kidney impairment in patients with psoriasis [8–10]. Accumulating evidence indicates that patients with various subtypes of psoriasis may develop IgAN, which can be seen in psoriasis vulgaris, pustular psoriasis, psoriatic arthritis, and erythrodermic psoriasis [11–16]. Furthermore, psoriasis may elevate the risk of IgAN [17]. Notably, a markedly increased likelihood of developing IgAN is observed among patients with moderate‐to‐severe psoriasis [10]. These findings suggest that both conditions have a potential pathophysiology in common. The underlying mechanisms linking psoriasis and IgAN have not been well elucidated. The pathogenesis of psoriasis is relatively complex. It involves multiple aspects, including immunity, inflammation, cell proliferation and apoptosis, microvascular abnormalities, neuropsychiatric factors, and neurotransmitters within a multigene genetic background. The interleukin (IL)‐23/T‐helper (Th)‐17 axis is thought to play a central role in its development [18]. IgAN is primarily defined by the deposition of IgA‐containing immune complexes in the glomeruli, leading to glomerular inflammation and potential progression to ESRD [19]. Research has confirmed that Gd‐IgA1 is a key effector molecule in the pathogenesis of IgAN [20]. Its pathogenesis comprises a complex interplay of genetic, immune, and environmental factors [19]. Both psoriasis and IgAN exhibit dysregulation of immune responses, characterized by the overproduction of inflammatory cytokines and immune mediators [18, 20]. For instance, immune dysregulation in psoriasis is primarily driven by the activation of Th17 cells, which play a pathogenic role in IgAN [19]. Therefore, we speculate that the intricate interplay between genetic predisposition, environmental factors, and immune system dysregulation contributes to the development of IgAN in individuals with psoriasis.

Genetic studies employing mendelian randomization (MR) have suggested a causal association between psoriasis vulgaris and increased risk of IgAN, highlighting shared genetic susceptibility and immune pathways [17]. Transcriptomic analyses reveal overlapping immune and inflammatory gene expression profiles, including key mediators such as IL‐1β, CXCL9, CCL4, and MMP1, which may serve as potential biomarkers or therapeutic targets for linking the two diseases [17]. Furthermore, single‐cell RNA sequencing meta‐analyses elucidate disease‐specific chemokine receptor interactions and immune cell migration patterns common to psoriasis and IgAN, providing insights into the mechanisms of immune cell recruitment and tissue infiltration in both the skin and kidney [21]. These findings support the concept that psoriasis and IgAN may represent interconnected immune‐mediated conditions with overlapping pathogenic pathways.

In summary, the coexistence of psoriasis and IgAN suggests shared immunopathogenic mechanisms involving genetic susceptibility, cytokine dysregulation, and immune complex‐mediated inflammation. Understanding these interrelationships is critical for early recognition, accurate diagnosis, and the development of targeted therapeutic strategies. This review aims to systematically summarize the current knowledge on the pathophysiological mechanisms and clinical manifestations of psoriasis complicated by IgAN, integrating recent advances in molecular and clinical research to provide a comprehensive perspective on their interplay and management.

## 2. Close Association Between Psoriasis and IgAN

### 2.1. The Incidence and Epidemiological Characteristics of IgAN in Patients With Psoriasis

Numerous studies have identified psoriasis as a distinct risk factor for CKD and glomerulonephritis (GN) [4, 5, 22, 23]. Among the diverse types of GN associated with psoriasis, IgAN is recognized as the most common and has the potential to advance to CKD and ESRD. A population‐based cohort study revealed that individuals with psoriasis face a significantly heightened risk of developing both CKD and GN, with the risk amplifying in accordance with the disease severity [22]. Furthermore, patients experiencing moderate‐to‐severe psoriasis demonstrated a greater propensity for the onset of IgAN, and this risk was statistically significant (HR 4.75, 95% CI 1.92–11.76) [10]. To be noted, the excess risk of IgAN associated with moderate‐to‐severe psoriasis was observed to be 1 in every 8888 individuals [10]. However, there have been limited epidemiological data on the relationship between psoriasis and IgAN. Therefore, there is an urgent need to conduct large‐scale multicenter cohort studies based on populations to further explore the epidemiological characteristics of both.

Beyond dedicated cohort studies, an important and yet largely overlooked source of evidence lies in the existing clinical trials for psoriasis. A review of major phase III randomized controlled trials of biologic therapies for psoriasis—including those targeting IL‐17A, IL‐23, and TNF‐α—reveals that urinalysis findings, such as proteinuria or hematuria, are not routinely reported as safety or efficacy endpoints. Neither baseline screening nor longitudinal monitoring of urinary abnormalities is consistently required or documented in these trials. This represents a significant knowledge gap and a missed opportunity. While these trials were not designed to detect renal outcomes, the absence of systematic urinary testing means that a potential signal of asymptomatic IgAN or other glomerulopathies may have been overlooked. Therefore, we propose that future psoriasis trials, particularly those evaluating novel immunomodulatory agents, should incorporate simple, cost‐effective urinary tests (e.g., dipstick analysis and urine albumin‐to‐creatinine ratio) at baseline and during follow‐up. Such measures would help determine whether subclinical renal involvement occurs in trial populations and could provide early safety signals relevant to the psoriasis–IgAN connection.

A retrospective study involving 90 patients with IgAN secondary to psoriasis revealed that those with severe psoriasis had a higher prevalence of impaired renal function, indicated by an estimated glomerular filtration rate (eGFR) below 60 mL/min/1.73 m^2^ and elevated proteinuria levels compared to patients with mild‐to‐moderate psoriasis [16]. Moreover, patients with severe psoriasis exhibited more pronounced pathological features such as tubular atrophy/interstitial fibrosis (T) lesions and crescentic lesions (C2, crescents in one‐fourth or more of glomeruli), which are markers of aggressive renal injury [16]. Importantly, during a median follow‐up of nearly 3 years, a significantly greater proportion of patients with severe psoriasis progressed to ESRD compared to those with less severe skin disease, underscoring the impact of psoriasis severity on the renal prognosis [16].

Moreover, the relationship between different types of psoriasis and the risk of IgAN appears to vary. MR analysis has shown that patients with psoriasis vulgaris and arthropathic psoriasis are at a notably increased risk for developing IgAN, with odds ratios (OR) of 1.040 and 1.081, respectively [17]. This indicates that specific subtypes of psoriasis may be more closely associated with renal disease than others, suggesting a potential differential pathophysiological mechanism at play.

### 2.2. Shared Genetic Basis Between Psoriasis and IgAN

MR analyses have recently provided compelling genetic evidence supporting a shared etiological link between psoriasis—particularly psoriasis vulgaris and psoriatic arthritis—and IgAN. A pivotal study employing MR methodology demonstrated that individuals with psoriasis vulgaris have a modestly increased risk of developing IgAN, with an odds ratio of 1.040 (95% CI 1.005–1.076, *p* = 0.026), while those with arthropathic psoriasis exhibit an even higher risk (OR = 1.081, 95% CI 1.040–1.124, *p* < 0.01) within European populations [17]. This genetic association underscores the possibility that common pathogenic pathways underlie these two clinically distinct conditions. Transcriptome analyses further identified a set of 12 crosstalk genes, including CXCL9, IL‐1β, CCL4, and MMP1, which are differentially expressed in both psoriasis and IgAN. Functional annotation of these genes revealed their predominant involvement in immune and inflammatory responses, suggesting that dysregulated immune signaling and chronic inflammation are central to the pathogenesis of both diseases [17].

Protein–protein interaction network analyses highlighted CXCL9, IL‐1β, CCL4, and MMP1 as hub genes with significant diagnostic potential, indicating their importance as molecular biomarkers and potential therapeutic targets for the comorbidity of psoriasis and IgAN. The MR findings are clinically relevant, emphasizing that clinicians should maintain heightened vigilance for renal dysfunction in psoriasis patients, especially those with psoriasis vulgaris and psoriatic arthritis, due to their genetically increased susceptibility to IgAN [17]. Notably, while psoriasis showed a genetic correlation with IgAN, other inflammatory skin diseases such as atopic dermatitis and acne did not demonstrate a significant causal relationship with IgAN in MR studies, highlighting the specificity of the psoriasis‐IgAN genetic link [24].

Taken together, these genetic and transcriptomic data provide a robust framework supporting a shared molecular and immunopathogenic basis between psoriasis and IgAN, mediated through key inflammatory chemokines and cytokines. This insight not only advances our understanding of the overlapping disease mechanisms but also paves the way for precision medicine approaches targeting these shared pathways in patients affected by both conditions.

### 2.3. Factors Affecting Epidemiological Data

There are a myriad of factors that may affect epidemiological data, including drug treatment, infections, environmental influences, and the immune status of patients. Biologic therapies, such as tumor necrosis factor (TNF) inhibitors and IL inhibitors, have revolutionized the treatment landscape for psoriasis, leading to improved patient outcomes and potentially altering the disease course. These therapies can modulate immune responses, thus impacting the overall incidence and severity of psoriasis‐related comorbidities, including IgAN [25]. Infections, particularly those that are chronic or recurrent, can exacerbate psoriasis flares and may contribute to the development of associated renal conditions. For example, streptococcal infections have been linked to the onset of guttate psoriasis, which may subsequently influence the immune response and predispose individuals to renal complications like IgAN. Environmental factors, including climate, pollution, and lifestyle choices, also play a crucial role in the epidemiology of psoriasis. Studies have indicated that exposure to certain environmental triggers, such as smoking and excessive alcohol consumption, can worsen psoriasis and its complications, including kidney involvement. Furthermore, the immune status of individuals, particularly those with underlying autoimmune conditions, can significantly influence the incidence of both psoriasis and IgAN. Individuals with a compromised immune system or those receiving immunosuppressive therapies may experience altered disease manifestations and increased susceptibility to infections that can exacerbate their skin and renal conditions.

Understanding these multifaceted interactions is essential for developing effective treatment strategies and public health interventions aimed at reducing the burden of psoriasis and its associated comorbidities, including IgAN. As the epidemiological landscape continues to evolve, ongoing research is crucial to elucidate the complex relationships between these factors and their collective impact on disease incidence and management.

## 3. Cross‐Talk and Interaction of Immune Inflammatory Mechanisms

### 3.1. Immune Inflammatory Signaling Pathways and Molecular Mechanisms

#### 3.1.1. IL‐17 Pathway

Psoriasis is characterized by the activation of the immune system, particularly Th17 cells, which produce proinflammatory cytokines such as IL‐17A, IL‐17F, and IL‐22 [26]. These cytokines are instrumental in promoting keratinocyte proliferation and the inflammatory response seen in psoriatic lesions [26, 27]. The presence of IL‐17A in psoriatic skin has been shown to correlate with disease severity, underscoring its role as a therapeutic target in psoriasis management. Similarly, in IgAN, the dysregulation of the immune response, particularly involving Th17 cells, has been implicated in its pathophysiology [28–30]. It has been hypothesized that IL‐17 may stimulate the production of Gd‐IgA1 based on in vitro studies [31], suggesting a potential contribution to the pathogenesis of IgAN. Elevated levels of IL‐17A have been detected in patients with IgAN, contributing to mesangial cell proliferation and the deposition of IgA in the kidneys [32].

Furthermore, it has been proposed that an imbalance between regulatory T cells and Th17 cells may contribute to the pathogenesis and progression of IgAN [33]. Additionally, levels of serum and urinary IL‐17A are found to be elevated in individuals with IgAN compared to those in other nephropathies and control cohorts [34]. Consequently, the blockade of IL‐17A could be beneficial not only for treating psoriasis but also potentially for managing IgAN to a certain extent. Biologics that inhibit IL‐17A, such as secukinumab and ixekizumab, have been effective in achieving significant improvements in psoriasis severity scores [35]. Meanwhile, IL‐17 blockade may reduce proteinuria and improve renal function in IgAN, although further research is needed to establish the long‐term benefits and safety of such interventions [36]. Intriguingly, in a previous case report, urinary abnormalities that did not respond to corticosteroid treatment alone showed improvement after the commencement of secukinumab therapy, indicating the therapeutic potential of the IL‐17A inhibitor.

Overall, the IL‐17 pathway plays a critical role in the inflammatory processes underlying both psoriasis and IgAN. Understanding the mechanisms by which IL‐17 contributes to these diseases not only enhances our comprehension of their pathophysiology but also opens avenues for targeted therapeutic strategies that could mitigate inflammation and improve patient outcomes. The dual role of IL‐17 in promoting inflammation while also potentially modulating immune responses underscores the complexity of its function and the need for careful consideration in therapeutic approaches targeting this cytokine.

#### 3.1.2. TNF‐α Pathway

The TNF‐α signaling pathway plays a complex and dual role in the context of psoriasis and IgAN. On the one hand, TNF‐α inhibitors are widely utilized in the treatment of psoriasis, providing significant relief from the inflammatory symptoms and improving the quality of life for patients suffering from this chronic skin condition. These biologics work by blocking the action of TNF‐α, a proinflammatory cytokine that is elevated in psoriasis, thereby reducing inflammation, keratinocyte proliferation, angiogenesis, and the overall psoriatic plaque formation [37]. However, the use of TNF‐α inhibitors carries the risk of paradoxical reactions, where patients may develop or experience exacerbation of other autoimmune conditions, including IgAN. This paradoxical effect is particularly concerning, as TNF‐α is also implicated in the regulation of immune responses and renal function. TNF‐α can influence the pathogenesis of IgAN by promoting inflammatory processes that lead to renal damage [13]. Macroscopic hematuria, along with proteinuria, became evident following the commencement of anti‐TNFα therapy, indicating that the underlying IgAN may have been aggravated due to an increase in IgA1 deposition triggered by the TNFα inhibitor [13].

A recent study has demonstrated a relationship between the levels of circulating TNF‐α receptors and the clinical outcomes of IgAN [38, 39]. Consequently, increased levels of circulating TNF receptors have been suggested as potential early biomarkers indicative of renal progression in patients diagnosed with IgAN [38]. The latest study has reported that infliximab could play a role in the remission of rapidly progressive IgAN associated with Crohn’s disease [40]. It shows that anti‐TNF‐α therapy has the potential to facilitate the resolution of hematuria and renal insufficiency by suppressing the inflammatory processes within the renal tissues [40]. Consequently, further research is essential to elucidate the precise function of anti‐TNF‐α therapy in the context of IgAN. Some scholars postulate a similar diminished glycosylation of IgA1 molecules in individuals developing IgAN subsequent to the administration of anti‐TNF‐α agents [41]. Biologic therapeutic agents may be perceived by the human immune system as ’foreign’ and provoke an immune response. Various classes of immunoglobulins with distinct affinities were generated. The generation of specific IgA and IgM antibodies targeting TNF‐α blocking agents is not intrinsically linked to any reduction in efficacy nor to systemic and local reactions [42]. Therefore, antibodies aimed at the glycans of the heavy chains of TNF‐α blockers may cross‐react with glycans on IgA1 molecules, resulting in the formation of substantial immune complexes. These complexes may traverse the endothelial fenestrae in the glomerulus and deposit within the renal mesangium. Alternatively, aberrantly glycosylated IgA1 could interact with the antigenic epitopes of TNF blockers. In some manner, large polymeric IgA complexes are generated.

Therefore, while TNF‐α inhibitors are effective in managing psoriasis, their role in IgAN remains to be further elaborated. This highlights the necessity for careful patient monitoring and management strategies when employing these therapies. Clinicians must weigh the benefits of TNF‐α blockade against the risk of inducing or worsening IgAN, particularly in patients with preexisting renal conditions or those presenting with new renal symptoms during treatment. This duality in the role of TNF‐α underscores the complexity of inflammatory pathways in autoimmune diseases and the need for a tailored approach in treatment, balancing the efficacy in controlling psoriasis with vigilance for renal complications.

#### 3.1.3. JAK‐STAT Pathway

**Figure 1:**
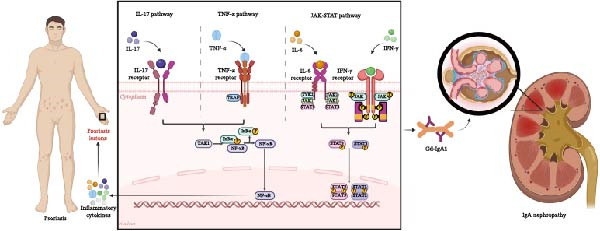
Immune inflammatory signaling pathways and molecular mechanisms between psoriasis and IgA nephropathy. Three pathways play significant roles in producing excessive Gd‐IgA1, including the IL‐17 pathway, TNF‐α pathway, and JAK‐STAT pathway.

The Janus kinase signal transducer and activator of transcription (JAK‐STAT) signaling pathway plays a central role in modulating immune cell proliferation, differentiation, and function, orchestrating both innate and adaptive immune responses. It regulates the activity of diverse immune cell types, including T cells, B cells, and dendritic cells (DCs), by mediating cytokine‐induced signaling that governs their development and effector functions [43]. Dysregulation of JAK‐STAT signaling has been implicated in the pathogenesis of various autoimmune and inflammatory diseases, including psoriasis and IgAN [44]. The JAK/STAT3 and JAK/STAT1 signaling pathways are significantly involved in the pathogenesis of psoriasis when activated by IL‐6 and interferon (IFN)‐γ, which are secreted by DCs and T‐lymphocytes [45] (Figure 1). The JAK‐STAT pathway regulates immune functions in psoriasis by modulating Th17 cell differentiation and dendritic cell activity, enhancing IL‐17 secretion and inflammatory responses [46]. Dysregulated JAK‐STAT activity in DCs promotes chronic inflammation and autoimmune responses [47]. Inhibition of this pathway reduces Th17 populations and cytokine production, highlighting its role in immune crosstalk and the immunopathology of psoriasis, making it a potential therapeutic target to restore the immune balance [48].

In patients with IgAN, multiple proinflammatory cytokines, such as IL‐6 and IFN‐γ, activate the JAK‐STAT pathway, thereby mediating inflammatory responses within the kidney. Studies have shown elevated phosphorylation of STAT proteins, particularly STAT1 and STAT3, in peripheral blood mononuclear cells (PBMCs) and renal tissues of IgAN patients compared to healthy controls, indicating a heightened baseline activation state of this signaling cascade [49]. Immunofluorescence and transcriptomic analyses reveal increased expression of JAK2 and phosphorylated STAT1 and STAT3 in glomerular mesangial cells, endothelium, and tubulointerstitial compartments, correlating with disease severity and proteinuria levels [49, 50]. The activation of STAT3 in particular promotes mesangial cell proliferation and extracellular matrix accumulation, which are key drivers of glomerulosclerosis and tubulointerstitial fibrosis, ultimately leading to renal function decline. Moreover, the degree of STAT1 activation has been associated with the progression of kidney damage, as demonstrated by increased pSTAT1 staining correlating with reduced eGFR and worsening histopathological features over time [50]. Additionally, other study have indicated that the aberrant activation of STAT3 induced by IL‐6 leads to an excessive production of galactose‐deficient IgA1 [51]. Consequently, the STAT3 signaling pathway may serve as a novel therapeutic target for the disease‐specific treatment of IgAN [51]. This evidence collectively underscores the pivotal role of JAK‐STAT signaling in orchestrating immune cell infiltration, cytokine production, and fibrotic remodeling in IgAN, positioning it as a central pathway in the disease’s immunopathogenesis.

In conclusion, the JAK‐STAT signaling pathway is a pivotal molecular axis in the pathogenesis of both psoriasis and IgAN, particularly through its central role in immune cell activation and regulation of inflammatory mediators. The accumulating evidence underscores that aberrant activation of this pathway is not only a hallmark of each disease individually but also a potential shared mechanistic link in patients presenting with comorbid psoriasis and IgAN. This convergence highlights the JAK‐STAT pathway as a critical molecular bridge connecting the immunopathological processes underlying these two seemingly distinct conditions.

#### 3.1.4. NF‐κB Pathway

The nuclear factor kappa B (NF‐κB) signaling pathway is a pivotal intracellular mechanism that orchestrates inflammatory and immune responses across a wide spectrum of physiological and pathological contexts. Upstream of NF‐κB activation, various proinflammatory cytokines and pattern recognition receptors (PRRs) play pivotal roles in initiating the signaling cascade. TNF‐α, IL‐17, and IL‐1β are potent activators of NF‐κB through their respective receptors, which recruit adaptor proteins and kinases, leading to the phosphorylation and degradation of IκBα [52, 53]. Experimental models using lipopolysaccharide (LPS) or ovalbumin have demonstrated that NF‐κB activation correlates with increased expression of inflammatory cytokines, such as IL‐1β, IL‐6, and TNF‐α, and enhanced immune cell infiltration, underscoring the central role of NF‐κB in driving systemic and local inflammation [54]. In the context of psoriasis, the IL‐17/IL‐23 axis, which is regulated by NF‐κB signaling, mediates skin inflammation by activating downstream molecules such as ACT1, TRAF6, and TAK1, leading to NF‐κB phosphorylation and nuclear translocation in keratinocytes and macrophages. This activation promotes the secretion of proinflammatory cytokines and chemokines that recruit immune cells, perpetuating the inflammatory cycle [55]. Moreover, NF‐κB activation in DCs is essential for their maturation and cytokine production, including IL‐6, IL‐12, and TNF‐α, which are crucial for T‐cell activation and the maintenance of immune imbalance observed in psoriasis [56]. The activation of NF‐κB also extends to macrophages and monocytes, where it regulates inflammasome activation, cytokine release, and cell survival, thereby influencing the inflammatory milieu and disease progression [57]. In psoriatic skin, keratinocyte‐derived transglutaminase 2 (TG2) activates NF‐κB, leading to upregulation of chemokines such as CCL20, which recruits IL‐17‐producing CCR6 + γδT cells and neutrophils, further amplifying inflammation [58]. This NF‐κB‐driven cytokine milieu sustains the activation and proliferation of T cells, including Th17 subsets, which secrete IL‐17, a key cytokine in psoriasis pathogenesis. Additionally, NF‐κB signaling contributes to the activation of other immune cells, such as DCs and T cells, maintaining the immune dysregulation characteristic of psoriasis [55, 58].

The NF‐κB signaling pathway plays a pivotal role in mediating local inflammatory responses within the glomerulus, particularly through its activation in mesangial cells and endothelial cells, which are critical components of the glomerular architecture. Upon activation, NF‐κB promotes the secretion of various proinflammatory cytokines and chemokines from these glomerular cells, thereby orchestrating a microenvironment conducive to immune cell infiltration and inflammation. In IgAN, mesangial cells activated via NF‐κB signaling secrete inflammatory mediators such as IL‐6, CXCL10, and CCL5, which contribute to mesangial proliferation and the recruitment of immune cells, amplifying glomerular injury [59]. This inflammatory cascade is further exacerbated by the deposition of IgA immune complexes that stimulate mesangial cells and endothelial cells to produce additional inflammatory factors, thus perpetuating a vicious cycle of inflammation and tissue damage [60]. The infiltration of macrophages, predominantly the proinflammatory M1 subtype, into the glomeruli is closely associated with mesangial proliferation and hematuria severity in IgAN patients, highlighting the critical role of NF‐κB‐mediated chemokine production in immune cell recruitment [61]. Similarly, the Chinese herbal formula Zhen‐Wu‐Tang exerts protective effects in IgAN by promoting exosome‐mediated inhibition of NF‐κB/NLRP3 signaling in mesangial cells, thereby reducing inflammation and proteinuria [62]. And the Yi‐shen‐hua‐shi granules influence immune responses and mitigate inflammatory injury through the ALG3/PPARγ/NF‐κB signaling pathway in the management of IgAN [63]. Furthermore, NF‐κB activation facilitates the amplification of IgA immune complex‐mediated inflammatory responses by enhancing the expression of adhesion molecules and chemokines that attract and activate immune cells within the glomerulus. This amplification is critical in the progression of IgAN, where immune complex deposition triggers sustained NF‐κB‐driven inflammation, leading to glomerular injury and fibrosis [64]. In addition to immune cell recruitment, NF‐κB activation in glomerular cells also modulates the expression of fibrogenic factors such as TGF‐β1, which links inflammation to fibrotic remodeling of the glomerulus, further contributing to the disease progression [65, 66]. Both in vivo and in vitro studies have indicated that TLR4/MyD88/NF‐κB pathway activation leads to Gd‐IgA1 overproduction, which is inhibited by TLR4 inhibitors [67]. Besides, tetrandrine suppresses the growth of mesangial cells that was stimulated by enzymatically deglycosylated human IgA1 through the IgA receptor/MAPK/NF‐κB signaling pathway [68].

Collectively, these findings underscore the central role of NF‐κB in mediating local glomerular inflammation by promoting inflammatory cytokine secretion from mesangial and endothelial cells and amplifying IgA immune complex‐induced inflammatory responses, highlighting NF‐κB as a promising therapeutic target in IgAN complicated with psoriasis.

### 3.2. The Relationship Between Aberrant Glycosylation of IgA1 and the Immune Response in Psoriasis

The aberrant glycosylation of IgA1 has emerged as a significant factor in the pathogenesis of IgAN [69]. A crucial mechanism underlying this phenomenon involves the downregulation of specific glycosyltransferases, notably GALNT12 and C1GALT1C1, which are responsible for the proper glycosylation of IgA1 [70, 71]. GALNT12 encodes a polypeptide N‐acetylgalactosaminyltransferase that initiates O‐glycosylation, while C1GALT1C1 encodes a core 1 β1,3‐galactosyltransferase essential for the addition of galactose to the O‐glycans on IgA1 [72]. The presence of Gd‐IgA1 in the circulation is associated with the formation of immune complexes that can deposit in the glomeruli, triggering inflammatory responses and contributing to kidney damage [73]. The accumulation of Gd‐IgA1 in the kidneys leads to the formation of immune complexes that activate mesangial cells, resulting in glomerular inflammation and injury. This process is mediated by the binding of Gd‐IgA1 to IgG autoantibodies, which can enhance the inflammatory response through the activation of complement pathways and recruitment of inflammatory cells [74, 75]. The clinical manifestation of this immune complex deposition often include hematuria and proteinuria, which are indicative of glomerular damage.

Abnormal glycosylation is significantly implicated in the pathogenesis of psoriasis, primarily through its regulatory effects on certain proteins, including the IL‐23 receptor, which is essential for the differentiation of Th17 cells and the subsequent development of psoriasis [76]. Glycans and glycan‐binding proteins serve to activate immune cells and stimulate the proliferation of epidermal cells through a variety of mechanisms. Glycosylation has direct implications for maturation, transport between the endoplasmic reticulum and Golgi apparatus, surface expression on the plasma membrane, and the signal transduction pathways mediated by receptors involved in this process [76]. Furthermore, the inflammatory milieu in psoriasis, marked by elevated cytokine levels, such as IL‐17 and IL‐6, further exacerbates the production of Gd‐IgA1, thereby contributing to the pathogenesis of both psoriasis and IgAN [31, 77]. Mechanistically, the expression of C1GALT1 and Cosmc was notably diminished in cells treated with IL‐17 compared to those in the control group [31]. It is worth mentioning that abnormal expression or activity of C1GALT1 and Cosmc leads to aberrant protein glycosylation [31].

Besides, Saso et al. [78] investigated the association between psoriasis and glycosylation, revealing that individuals suffering from psoriatic arthritis demonstrate elevated serum reactivity to concanavalin A, a compound that selectively binds to glycan chain structures, in contrast to healthy controls. This heightened response shows a correlation with inflammatory markers [78]. Although the study did not pinpoint specific glycoforms, it laid the groundwork for understanding the interplay between psoriasis and glycosylation. Advancements in experimental methodologies have revealed distinct serum N‐glycan profiles in psoriasis patients. Additionally, research on tissue N‐glycans indicates that core fucosylation may influence psoriasis pathogenesis, as FUT8 expression is notably higher in psoriasis keratinocytes, correlating with lesion severity and facilitating epidermal growth factor receptor (EGFR), overactivation, and keratinocyte hyperproliferation, contributing to the phenotypic manifestations of psoriasis [79].

Future research should focus on larger, more comprehensive studies to further explore the connection between psoriasis and glycosylation and the relevant mechanism. Understanding these mechanisms opens avenues for targeted therapeutic strategies aimed at correcting glycosylation defects and modulating the immune response, potentially improving outcomes for patients suffering from both psoriasis and IgAN. The relationship between aberrant IgA1 glycosylation and the immune response in psoriasis suggests a complex interplay between mucosal immunity and systemic inflammation.

### 3.3. Systemic Inflammation and Renal Injury

Chronic systemic inflammation is a significant contributor to the pathophysiology of various renal diseases, particularly CKD and IgAN. Systemic inflammatory mediators, such as cytokines and chemokines, can profoundly impact the integrity of the glomerular filtration barrier, leading to alterations in kidney function. Cytokines like TNF‐α and IL‐6 have been shown to promote glomerular inflammation and fibrosis, ultimately resulting in a decline in the GFR [80, 81]. The persistent activation of inflammatory pathways can lead to a vicious cycle where renal injury perpetuates further inflammation, contributing to the progression of CKD and other renal pathologies. In patients with IgAN, the deposition of galactose‐deficient IgA1 in the mesangial area triggers a local inflammatory response characterized by the activation of mesangial cells, leading to the production of proinflammatory cytokines and chemokines, which exacerbate renal injury and dysfunction [82, 83].

**Figure 2:**
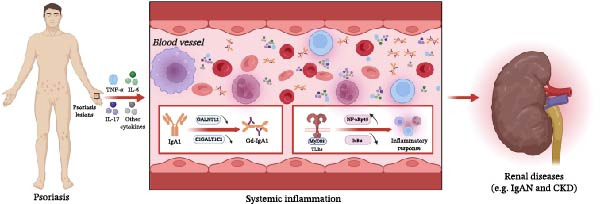
The mechanism of systemic inflammation mediated the exacerbation of CKD and IgAN by psoriasis. Inflammatory skin releases cytokines, including IL‐17, TNF‐α, IL‐6, and IL‐23, into the bloodstream, causing systemic inflammation. It enhances Gd‐IgA1 production and renal immune deposition while activating TLR‐mediated kidney injury, thereby exacerbating IgAN and CKD.

Furthermore, the relationship between skin lesions in psoriasis and renal impairment has been documented, with studies indicating that the inflammatory response associated with psoriasis skin lesions may precipitate renal damage [84]. Specifically, the inflammatory reaction to psoriatic lesions can elevate the concentrations of inflammatory mediators in both the serum and renal tissues. These mediators might instigate inflammation in the renal tubular epithelial cells and mesangial cells, consequently leading to renal impairment. Furthermore, the inflammatory response elicited by psoriatic lesions could activate Toll‐like receptors (TLRs) and MyD88 (myeloid differentiation factor 88) signaling pathways, thereby enhancing the expression of NF‐κB‐related proteins (notably NF‐κBp65) while diminishing IκBα protein levels. These alterations may further fuel the inflammatory response and intensify renal injury [85] (Figure 2). This suggests that systemic inflammation associated with skin conditions may extend its detrimental effects to the kidneys, highlighting the need for a comprehensive understanding of the interplay between chronic inflammation and renal health [84].

In addition to direct inflammatory effects, chronic inflammation can also influence kidney function through systemic mechanisms. For example, the presence of chronic low‐grade inflammation in CKD patients has been associated with increased cardiovascular risk, which further complicates the management of renal diseases [86]. The interplay between inflammation and cardiovascular health can create a feedback loop that exacerbates renal dysfunction as cardiovascular disease can lead to renal hypoperfusion and ischemia, worsening kidney injury. Moreover, the activation of the renin–angiotensin–aldosterone system (RAAS) in response to systemic inflammation can contribute to hypertension and fluid retention, further impairing renal function [87].

Beyond the canonical inflammatory pathways discussed above, emerging evidence indicates metabolic dysregulation, particularly insulin resistance, as a potential common denominator linking the cutaneous inflammation of psoriasis to the progression of renal injury in IgAN. Insulin resistance, often quantified by the triglyceride–glucose (TyG) index, has been shown to drive endothelial dysfunction, oxidative stress, and heightened inflammatory responses [88]. In the context of psoriasis, observational studies have demonstrated a stable and robust positive association between TyG‐related indicators and the disease presence [89]. Furthermore, the TyG index has been shown to independently predict adverse renal outcomes, including progression to ESRD, in patients with CKD [90]. Together, these observations suggest that insulin resistance may act as an independent driver or amplifier of the psoriasis–IgAN nexus, complementing the classical immune‐inflammatory pathways discussed earlier. Future studies should explore whether targeting insulin resistance could mitigate both skin and renal manifestations in this comorbid population.

The correlation between the activity of skin lesions and renal injury is particularly relevant in the context of psoriasis, where systemic inflammation is a hallmark of the disease. Studies have shown that patients with psoriasis are at an increased risk for developing CKD [91]. This relationship underscores the importance of monitoring renal function in patients with chronic inflammatory conditions as early intervention may mitigate the progression of renal disease. Furthermore, therapeutic strategies aimed at reducing systemic inflammation, such as the use of anti‐inflammatory agents or biologics, may hold promise in preserving the renal function in these patients.

In conclusion, systemic inflammation plays a critical role in the pathogenesis of renal diseases, particularly in CKD and IgAN. The impact of inflammatory mediators on the glomerular filtration barrier and the interplay between chronic inflammation and renal health highlight the need for a holistic approach to managing patients with chronic inflammatory conditions. Addressing systemic inflammation may not only improve renal outcomes but also enhance the overall quality of life for the affected individuals.

### 3.4. Chronic Tonsillitis and Immune Activation

The palatine tonsils are critical components of the immune system, acting as sites for immune activation and response. Psoriasis and IgAN are classified as tonsillar focal diseases (TFDs), where the tonsils serve as the primary origin of the pathology, resulting in reactive organic or functional harm to organs that are located far from the tonsils. Certain individuals diagnosed with psoriasis vulgaris may observe a worsening of their skin condition in conjunction with upper airway inflammation. Reports indicate that these individuals tend to achieve more favorable results following a tonsillectomy [92]. Additionally, the tonsillar tissue in patients with IgAN exhibits histological changes that correlate with the severity of renal lesions, indicating a potential link between chronic tonsillitis and renal pathology [93]. A recent observational cohort study utilizing a large health administrative dataset that included over 4 million individuals indicates a correlation between chronic tonsillitis and an increased risk of developing IgAN in the Japanese population [94]. In a cohort study, patients undergoing tonsillectomy experienced significant reductions in proteinuria and improvements in renal function post‐surgery, highlighting the potential therapeutic role of tonsillectomy in managing IgAN [83, 95]. A substantial body of clinical observational research, including randomized prospective trials, has demonstrated the significant effectiveness of tonsillectomy in the management of TFDs, which has consequently led to its endorsement in recent clinical guidelines [96, 97]. However, it is critical to contextualize these findings. The robust evidence supporting tonsillectomy for IgAN, including randomized prospective trials and guideline endorsements, originates predominantly from East Asian populations, particularly Japan, and shows greater efficacy in younger patients [83]. Consequently, tonsillectomy is not recommended as a standard or definitive treatment for IgAN in major international guidelines for all populations, and its use in non‐Asian or older cohorts remains controversial due to a lack of high‐quality evidence.

**Figure 3:**
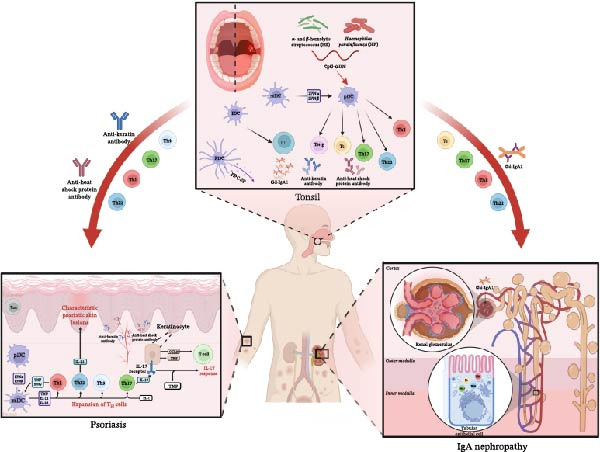
The tonsil‐centered shared pathogenesis of psoriasis and IgAN. Tonsil‐derived T cells migrate to the skin, where they interact with keratinocytes and release cytokines such as IL‐17 and TNF‐α. This triggers epidermal inflammation and hyperplasia, leading to the formation of psoriatic lesions. Simultaneously, autoantibodies produced in the tonsils target the skin, exacerbating local inflammation. Tonsillar immune dysregulation drives B cells to produce Gd‐IgA1, which forms circulating immune complexes that deposit in the glomerular mesangium, initiating nephritis. Concurrently, tonsil‐derived T cells infiltrate the kidneys directly, contributing to renal tissue damage.

The pathogenesis of TFDs has not been fully clarified yet. Recently, Yasuaki Harabuchi et al. have proposed the concept of tonsil‐induced autoimmune/inflammatory syndrome (TIAS) as a potential common mechanism underlying several diseases, and immunological research has uncovered a shared underlying mechanism for TIAS [98, 99]. TIAS posits that an exaggerated immune response instigated by the tonsils, typically provoked by prevalent bacteria such as α‐ and β‐hemolytic streptococcus (HS) and *Haemophilus parainfluenzae* (HP), which are normally found in the oral cavity, can become dysregulated [99]. This immune response is also characterized by the involvement of unmethylated DNA sequences (CpG‐ODN) that are prevalent across various bacterial species. In response to these bacteria or CpG‐ODN, populations of tonsillar T cells, including Th17, Th22, Th1, and cytotoxic T cells, undergo proliferation. Concurrently, tonsillar B‐cell populations that react to these stimuli generate antibodies targeting epidermal keratins and heat shock proteins or produce aberrant IgA1 [99]. These activated tonsillar T‐cell populations and antibodies subsequently enter the systemic circulation, allowing them to migrate to the kidneys or skin [99]. Upon arrival at these organs, they contribute to tissue damage, facilitated by the actions of various cytokines. IgAN and psoriasis, which exemplify disorders associated with TIAS, all share this underlying pathogenic mechanism, in which the tonsils link to each other (Figure 3).

Thus, chronic tonsillitis represents a significant factor in the dysregulation of immune responses. The interplay between chronic inflammation in the tonsils and systemic autoimmune conditions like IgAN and psoriasis illustrates the need for a comprehensive understanding of mucosal immunity and its implications for disease management. The potential benefits of tonsillectomy in ameliorating the symptoms of IgAN and psoriasis further emphasize the importance of considering chronic tonsillar inflammation in the therapeutic strategies for these conditions.

Together, the epidemiological, genetic, transcriptomic, and mechanistic evidence reviewed in Sections 2 and 3 consistently supports a clinically relevant link between psoriasis and IgAN, yet each line of evidence carries inherent limitations, such as possible surveillance bias, lack of longitudinal validation, or population‐specific findings. Table 1 summarizes the key studies discussed above, highlighting their main contributions and respective shortcomings. This synopsis reinforces the existence of shared immunoinflammatory pathways and underscores the pressing knowledge gaps—particularly the absence of routine renal screening in psoriasis trials and the scarcity of international registry data—that need to be addressed in future research.

**Table 1:** Summary of key evidence linking psoriasis and IgAN.

Study/evidence	Type	Main finding	Limitations
Grewal et al. [10]	Population cohort	HR 4.75 for IgAN in moderate‐to‐severe psoriasis	Possible surveillance bias; single cohort
Chen et al. [17]	MR + transcriptomics	Causal genetic link; 12 hub crosstalk genes	Horizontal pleiotropy; European‐only MR
He et al. [16]	Retrospective case series, *n* = 90	Severe psoriasis → worse renal outcomes	Small sample; single‐center; selection bias
Lin et al. [31]	In vitro	IL‐17 stimulates the production of Gd‐IgA1 in Dakiki cells	In vitro only; cell line model
Uriol‐Rivera et al. [36]	Case report	Secukinumab improved refractory proteinuria	Single case; no controls
Segawa et al. [13]	Case report	New‐onset IgAN after infliximab	Single case; no causal inference
Tao et al. [49]	Cross‐sectional	Elevated pSTAT1/3 in IgAN kidney tissue	Cross‐sectional; no longitudinal data
Yamada et al. [51]	Preclinical	STAT3 activation → Gd‐IgA1 overproduction	In vitro model
Nakayama et al. [94]	Administrative cohort	Chronic tonsillitis → IgAN risk (Japanese)	Japanese population only; administrative data
Harabuchi [99]	Review/framework	TIAS framework proposal	Proposed mechanism; not yet validated
Rahman et al. [21]	scRNA‐seq meta‐analysis	Shared chemokine signatures	Heterogeneous dataset sources; limited functional validation

## 4. Conclusion and Prospects

The coexistence of psoriasis and IgAN represents a multifaceted clinical challenge driven by complex interactions among immune dysregulation, genetic susceptibility, and inflammatory mediators. This intricate interplay underscores the need for a conceptual framework that transcends isolated disease perspectives—highlighting shared pathways such as IL‐17, TNF‐α, JAK‐STAT, and NF‐κB, while acknowledging distinct organ‐specific manifestations.

Moving beyond descriptive association, several critical questions warrant priority investigation. First, does the severity or subtype of psoriatic inflammation directly correlate with serum levels of Gd‐IgA1 or with the risk of progressive kidney disease? Second, can therapeutic targeting of shared pathways (e.g., IL‐17 or JAK inhibition) not only ameliorate psoriasis but also prevent or reverse early IgAN? Third, what are the precise cellular and molecular mechanisms by which tonsillar dysbiosis or infection triggers both cutaneous and renal inflammation? Answering these questions will be pivotal for moving from descriptive association to mechanism‐based, personalized management.

To overcome the limitations of heterogeneous, single‐center, or registry‐free observational data, future research should prioritize the establishment of large‐scale, multicenter, and ideally international patient registries dedicated to psoriasis‐associated IgAN. Such registries would enable systematic collection of standardized clinical data, laboratory parameters (including serial measurements of proteinuria, eGFR, and Gd‐IgA1 levels), histopathological findings from renal biopsies, detailed records of psoriasis treatments (e.g., biologics, phototherapy, and conventional systemic agents), and long‐term follow‐up outcomes. By integrating these multidimensional data, registries would facilitate an accurate estimation of the true incidence and prevalence of IgAN across different psoriasis subtypes, identification of independent risk factors for renal progression, and comparative assessment of therapeutic effectiveness and safety. Ultimately, high‐quality registry‐based evidence will be essential for developing robust, evidence‐based clinical guidelines and for designing future interventional trials in this understudied comorbidity.

From a clinical perspective, we propose a preliminary framework for patient management. Clinicians should maintain a low threshold for renal screening (urinalysis, serum creatinine, and eGFR) in patients with long‐standing, moderate‐to‐severe, or arthropathic psoriasis. Future clinical trials in psoriasis should incorporate routine urinalysis to detect subclinical renal involvement. While tonsillectomy may be considered in a select subset of young East Asian patients with refractory IgAN and chronic tonsillitis, it is not recommended as a global standard of care. The choice of biologic therapy should be individualized, weighing the robust efficacy of IL‐17 or JAK inhibitors against the potential for paradoxical IgAN reactions reported with certain TNF‐α blockers. Advancing our understanding of the psoriasis–IgAN nexus will ultimately translate into improved prognostic accuracy, targeted therapeutic strategies, and a better quality of life for affected patients.

## Author Contributions

**Zijie Tang**: conceptualization, investigation, writing – original draft. **Jintong Wu and Meihan Dong**: writing – original draft. **Chengxin Li and Rui Wang**: conceptualization, supervision, writing – review and editing.

## Acknowledgments

The authors thank Chinese People’s Liberation Army (PLA) General Hospital for their support of this work and the reviewers for allowing us to make improvements to the manuscript.

## Funding

This study was supported by the National Natural Science Foundation of China (Grants 82504266 and 82273530).

## Disclosure

All authors contributed to the article and approved the submitted version.

## Conflicts of Interest

The authors declare no conflicts of interest.

## Data Availability

No new data were generated for this manuscript.
